# AIOps Architecture in Data Center Site Infrastructure Monitoring

**DOI:** 10.1155/2022/1988990

**Published:** 2022-07-20

**Authors:** Wei Dong

**Affiliations:** IBM, Shenzhen 518000, China

## Abstract

AIOps (artificial intelligence for IT operations) has been growing rapidly in recent years. However, it can be seen that the vast majority of AIOps applications are implemented in the IT domain. In contrast, there are few applications in the data center infrastructure domain. Many real-world practices show that a working architecture or algorithm cannot be directly replicated from other domains due to completely different business scenarios and different data characteristics. In this paper, an AIOps architecture for the data center infrastructure monitoring domain is presented. A proven working architecture is given in terms of core modules, such as technical architecture, machine learning algorithms, big data, and business applications, and details are designed in practice. This paper focuses on the technical part and not on each part of the architecture work. In other words, NFRs (nonfunctional requirements), such as performance, availability, manageability, and security, will not be discussed in this paper.

## 1. Introduction

AIOps and APM (application performance management IT infrastructure management) systems have evolved rapidly in the last 5 years. However, AIOps technology has not made some significant progress in data center infrastructure management. It seems that many reasons have contributed to this problem. In particular, the first two reasons, the relatively small market size and the lack of AIOps capabilities of most DCIM vendors, are the main factors. On the other hand, technology is another challenge. For example, an algorithm may work well in one particular scenario; however, that algorithm may not perform well in another scenario because the data and features are different. Therefore, the design needs to start from the architecture. In this paper, we propose a complete and detailed AIOps architecture which is designed for data center on-site infrastructure monitoring. The mechanism and architecture design are described by the implementation and real-world performance of a number of different machine learning algorithms, using data center room temperature data as an example. The test data clearly demonstrate the effectiveness of this architecture. In addition, this architecture can be a useful reference for those who want to know how to implement machine learning algorithms in data center site monitoring systems.

## 2. Research Background

Typically, base station rooms and data center rooms have 3 characteristics that require high concurrency, high throughput, and other nonservice functions to be met when doing the architecture design. These 3 characteristics are as follows.

### 2.1. Large-Scale Equipment

Taking Shenzhen as an example, in terms of the number of base station rooms, there were 89,000 4G and 5G base stations in 2019. Assuming that there are 10 devices to be monitored, the size of the devices is 890,000 units. Furthermore, if a telecom operator has a 40% market share, the number of devices is expected to have a huge surge, possibly exceeding 356,000. You can see that the value of the number of devices is very large.

### 2.2. Various Equipment Types and Many Indicators in Different Equipment

**Figure 1:**
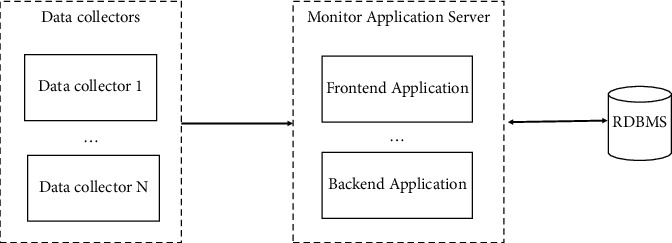
Architecture diagram.

It is possible to classify the facilities of data center rooms into the following 4 categories. Specifically, there are different equipment in different categories. Even the same facility supplier can produce so many different types of products in a particular category. As shown in Figure 1, the components include the following: power monitoring, environmental monitoring, security monitoring, and*∗* network monitoring (optional); the items include the following: power meters, distribution switches, UPS, diesel generators, distribution cabinets, PDUs, DC power supply systems, STS, and ATS battery packs; temperature and humidity, precision air conditioning, water leakage, server room air conditioning, fresh air units, haze, and harmful gases; CCTV, access control system, fire extinguishing system, lightning detection, and antitheft monitoring. Routers, switches, servers, and firewalls [1], as shown in Table 1.

### 2.3. High Sampling Frequency

In the power supply and environmental monitoring system, the FSU (field supervision unit) is a device that is mainly used for data acquisition and is designed to perform data acquisition [2]. Usually, it collects hundreds of devices every 100 ms or so, and the SC (supervision center) gets this data from the FSU periodically. In most cases, the sampling frequency of the SC is provided as a configuration. Given its value of 1 time/minute, the reported metrics data will be huge because there are many devices that make up many metrics. In addition, those alarm data or other necessary data, such as event data, should be included.

## 3. Materials and Methods

### 3.1. Technical Architecture Evolution

#### 3.1.1. All-in-One Architecture

In earlier years, the architecture of data center monitoring systems was a multi-in-one model [3]. Although logically divided into 3 layers, both front-end applications and back-end applications are tightly integrated in this architecture. Usually, the tendency is to use the same framework for all the development work, including the database; for example, all the application development was done with.Net or MFC + SQL Server around the year 2000.

Figure 1 shows the schematic of the all-in-one architecture.

Benefits are as follows:

1. Easy to deploy and operate the work.
2. In case of a low workload, this architecture can run well and meet business needs in terms of performance.

Disadvantages are as follows:

1. In general, scalability is its weakness. It is difficult for this architecture to accommodate fast-growing data, which is difficult for a vertically scaling server to handle.
2. Another problem is performance deterioration. As the amount of data grows, eventually, the amount of data will exceed a certain limit, and the performance of the database will gradually deteriorate. Problems such as unresponsiveness, latency, and even core downtime often occur posing a threat to the business operations.

#### 3.1.2. Big Data Architecture

**Figure 2:**
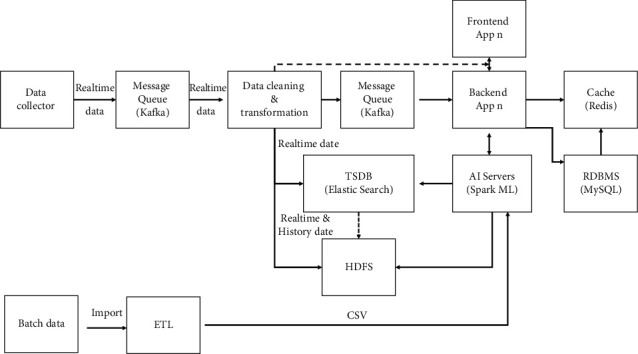
Architecture diagram.

After 2010, both big data and distributed technologies have become increasingly popular [4]. In particular, the decoupling of front-end applications and back-end applications is accompanied by the introduction of big data, caching, and other technologies in the architecture. The big data architecture is shown in Figure 2.

Benefits are as follows:

1. Spark, HDFS, Kafka, Redis, and MongoDB middleware are widely used in current technology solutions, enhancing throughput and alleviating performance bottlenecks
2. Thanks to the natural advantages of distributed and big data technologies, the response time of the reporting module is also greatly improved, especially when querying historical data [5]

Disadvantages are as follows:

It is not efficient enough compared to the progress of APM on AIOps. It lacks artificial intelligence techniques to automate some operational tasks, especially in anomaly detection and root cause analysis work

For example.

1. A large amount of configuration work is handled manually.
2. The event is usually not noticed until it occurs.
3. Although CEP (complex event processing) can raise some alerts in advance. Overall, the MTTD (mean time to detection) is still slow. As devices and applications are added, new problems are added, creating serious challenges for modules that have fixed rules centered on user experience.
4. Long MTTR (mean time to repair).

**Figure 3:**
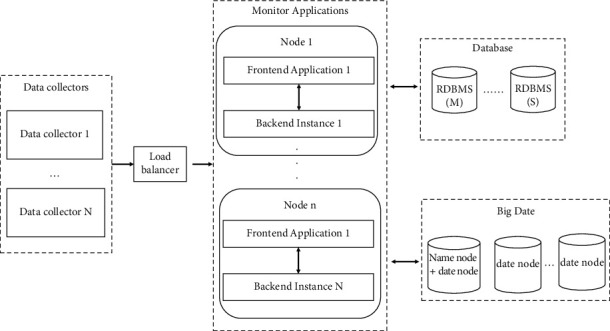
Big data architecture.

RCA (root cause analysis) depends entirely on the skills and experience of engineers, and its time cost varies from person to person. In general, the speed of problem-solving remains low, as shown in Figure 3.

#### 3.1.3. Artificial Intelligence + Big Data

The introduction of artificial intelligence technology can improve the quality of the product and increase productivity [6]. An architecture combined with AI can be a good solution for redundant work in certain scenarios and can also help in reducing MTTD and MTTR. The following is an AIOps architecture which consists of big data, AI, and microservices, as shown in Figure 2.

### 3.2. Architecture Design

(1) Mainly, the architecture is for the product. In other words, the architecture is the technical design of the product, including functional and nonfunctional features [7]. In general, an AIOps product needs to satisfy these requirements. First, it can be presented to run and delivered as a holistic solution. Second, it can be embedded directly into a web page or other 3rd application. Finally, it is also capable of being served entirely as a back-end application. The advantages of a product with such features are self-evident. Regardless of what the product is, as a specific solution or being integrated into other products, such a feature would be well suited to meet the flexibility requirements of the market.
(2) To address the problem of heterogeneous data access by third parties, a mapping table was designed in the database design work [8]. Since the reported data and the internal AIOps data may differ in terms of device types and metrics. A device mapping table is needed with a schema consisting of raw device type, raw metrics, internal device type, and metrics.
As shown in Tables 2 and 3.
(3) In the data storage section, different data are considered to have different usage frequencies, and these data are classified according to the access frequency. Specifically, hot data, warm data, and cold data are defined as having been accessed at least once in the last 24 hours, 1 month, and more than 1 month, respectively. Moreover, this feature is configurable. On the other hand, different data have different storage media. Accordingly, hot data are stored in Redis and Elasticsearch, warm data is stored in Elasticsearch, and cold data are stored in HDFS in the order given. In contrast, business data are stored in an RDBMS, such as MySQL.
Focusing on the feature that the writing frequency of the performance data is much higher than the reading frequency, the time series database Elasticsearch is adopted to store the performance data and alarm data.
(4) Processing and storing training data, prediction results, and real-time performance data in different systems. The reason is that we wish to avoid the performance of the system from being affected. Specifically, assuming that the collection, processing, and storage of real-time data are deployed on a single device, this device will become a single point of failure, as this data may be used to support concurrent requests from the business and processed by multiple back-end services, e.g., AI computing modules and back-end applications. Given the high system performance requirements of the AI module and back-end applications, two different Kafka themes are used to distribute real-time data to Elasticsearch and the HDFS, respectively. For example, both real-time data storage requirements and processing requirements need to be met. And generally, due to decoupling, these two requirements are implemented in two different systems. Therefore, if these two systems are designed to access the same storage medium at the same time, it will create a bottleneck in the system, and the situation will even deteriorate rapidly when dealing with large amounts of real-time data.
(5) To improve data throughput, Kafka + Spark streams were introduced. Compared with traditional MQ middleware, Kafka has higher performance in terms of reading/writing speed, throughput, and reliability. On the other hand, the Spark streams are definitely better than traditional ETL tools in real-time data processing.
(6) In the machine learning section, the following features are introduced with Python + scikit-learn.
  - Semi-automatic data annotation methods based on machine learning algorithms and manual corrections
  - Data import. For example, importing data through web pages or web services
  - Feature selection by expert experience
  - Algorithm management
  - Automatic optimization of hyperparameters
  - Model rolling optimization
In addition, for comparison, a deep learning algorithm LSTM is adopted for validation, and the Keras framework is used to implement LSTM.
(7) In terms of business service applications, business-function-oriented web services are proposed to combine artificial intelligence with business services. Also, these web services are provided on the web accordingly, such as real-time performance data prediction, alarm prediction, AI algorithms, feature management, and training data management. From a technical point of view, Spring Cloud + VUE + Nginx is used to decouple the back-end and front-end. The business services are based on Spring Boot and Spring Cloud microservices.
(8) For bulk data, an additional ETL toolset is provided to easily import data into HDFS or ES.

### 3.3. Main Service Flows

1. The sensor data collection application sends real-time data to the Kafka message queue.
2. The data cleaning and transformation module receives the message, subscribes to the relevant topics, and completes the data cleaning work, e.g., data anomaly detection and missing data processing [9]. In particular, the anomaly detection module will mark the type of data, whether it is normal or not. If the data is anomalous, then both the original data and the corrected data will be saved separately. Usually, these data are saved in Elasticsearch and the HDFS. Also, the data is forwarded to Kafka so that the back-end service can be notified immediately.
3. The back-end service gets the real-time data on the subject of Kafka and displays them through a web page. A line graph is proposed in which two lines are drawn, namely, the real-time data line and the predictive data line.
4. Thus, users can easily switch between different algorithm graphs so that the most suitable algorithm stands out naturally. (b) In addition, the back-end service will analyze the gap between real-time and predicted data and report alerts immediately or not, depending on the results of the analysis of dynamic thresholds.
5. To prevent the model drift problem, the hyperparameters of the AI model will be updated periodically based on the latest data [10]. Therefore, a configuration page is provided to set the frequency of cron jobs, specifying the time, such as hours, days, weeks, or months. For example, a job can be executed every 24 hours. The job will obtain the latest 7 *∗* 24 data to train the model. Given a device-specific metric, it will calculate and give predicted values for the next 24 hours. These values will be stored in the cache and RDBMS instead of the HDFS, which ensures the timeliness of the system interactions.
6. In addition to this, the commercial application, a java back-end service, also provides management and configuration modules such as device management, metrics management, algorithm management, and model management [11].

## 4. Results and Discussion

### 4.1. Technology Selection and Core Implementation

#### 4.1.1. Message Queue Kafka

Compared to other MQs, Kafka is able to handle larger volumes of data and can easily scale without data loss [12]. On the other hand, as a product of the big data ecosystem, Kafka is widely used in various projects. Therefore, it has proven to be stable. Last but not least, it is possible to easily decouple these different services using Kafka, thus enhancing the robustness of the system.

#### 4.1.2. Data Transformation Module

The main functions of the data transformation module are as follows:

1. Exception processing of historical data.
2. Usually, it is not necessary to handle exceptions to historical data immediately [13]. Various machine learning algorithms, such as IForest, KNN, DBScan, and the traditional fixed threshold, are used for exception detection. When the results of these algorithms are incorrect, one can also correct the results of the data labels.
3. It is also necessary to classify these data by anomaly type [14]. In general, these types of anomalous data are derived and different strategies are adopted to deal with them, as shown in Table 4.
4. Exception processing of real-time data. Considering the processing delay, relevant APIs are directly used to get fast results instead of using machine learning algorithms such as IForest, KNN, and DBSCAN [16].
5. Null and missing data are discarded and stored in the HDFS and Elasticsearch. As a compensating measure, prediction algorithms such as ARIMA, Holt-Winters, and LSTM are used to generate relevant new data to replace the original data.
6. Contextual anomalous data may be compared to normal data, such as “1,” “2,” “3”.! @#..., marks contextual exception data as an abnormal value. In addition, additional modifications are suggested, for example, replacing it with the latest data.
7. After the data processing work is finished, the original data and the processed data are stored in the database. This work can then be repeated in the same way when the model is updated with the latest new data [17].
8. Conversion of data types
9. Depending on the system requirements, the raw data need to be converted into internal data for AIOps, as mentioned in the data mapping section above. The raw data will be sent to Kafka. Then, this data will be processed by the ETL application [18]. As a result, the processed data will be distributed among ES, HSFS, and MySQL. Also, the processed data will be sent to Kafka to notify the back-end services to complete the real-time processing on time.
10. Technical Framework
11. The ETL application is implemented using Spark streaming and Scala, and the ETL application is treated as a standalone application. The reason why Scala and Kafka are used instead of other languages to interact with ES and MySQL is because Scala has a clear advantage in real-time processing (although Storm is better than Scala in terms of real-time processing in the internal tests conducted, the difference between Storm and Scala is small. Most importantly, Scala's ecosystem is better than Storm's ecosystem. In other words, it is a trade-off). In short, everything that is done is to increase the processing speed and reduce the complexity of the technology stack.

### 4.2. Data Storage

Data storage design is not an easy task. One needs to take into account the business realities and the goals of the project and try to give the best solution.

In this monitoring system, a TSDB is used to store performance data and alarm data. Besides, considering the response latency requirement and the different frequencies between read and write operations, a multitiered storage system was built, which includes TSDB, cache, RDBMS, and HDFS. First, there is the fact that the write time is much more than the read time. TSDB, such as InfluxDB or Elasticsearch, is a better choice compared to those traditional RDBMSs. On the other hand, MySQL is chosen as the RDBMS to store business data because it has excelled in traditional business data storage for the past 20 years. For data that are used frequently, it is stored not only in the RDBMS but also in a cache, such as Redis. For archived data and training data, HDFS is chosen because it is an inexpensive and reliable solution for storing big data.

#### 4.2.1. TSDB

Choosing a TSDB to store performance data is a natural choice. However, choosing which TSDB product to choose is not an easy task, as one needs to know exactly what one wants to have, and also needs to give comparative dimensions for these different TSDB products.

Mainly, investments were made in InfluxDB, ES, and OpenTSDB.

Although InfluxDB has better performance in either the standalone mode or distributed mode. However, since the distributed version of Influx is a paid software, the switch was quickly made to ES, which is a free distributed-oriented product with fairly high throughput and user-friendly queries. In addition, OpenTSDB was reviewed. However, as known, a few projects did choose OpenTSDB. Unfortunately, many of them abandoned OpenTSDB in favor of ES for various reasons, including some top companies.

#### 4.2.2. Storage Classification and Hierarchy

For business data, the following different business types are present: time-series data (performance data), alarm data, and business data.

These data have different frequencies of use. Therefore, it is necessary to make the frequency configurable to meet different requirements.

Hot data, stored in Redis and ES, are defined as having been accessed at least once in the last 1 week.

Hot data, stored in ES, are defined as having been accessed at least once in the last 1 month. Warm data consists mainly of time-series data, such as performance data and alarm data.

Cold data, stored in HDFS, are designed to store archived data or training data. It would be a better choice for storing data that has not been accessed for more than 1 month.

Finally, the business data are treated as a single type and MySQL is used to store it.

#### 4.2.3. Repeated Storage to Avoid SPOF (Single Point of Failure)

Assume that both data processing and data storage are installed on one node (server). Since a large amount of data may be reported at the same time, this node may become the SPOF of the system. The reasons are as follows: first, the data may be accessed by those front-end services or users at the same time, but it is also possible that the data are being processed by other services, for example, artificial intelligence applications, which is a time-consuming and high-throughput data access. Therefore, Kafka is used to forward the data to the ES and HDFS (or other storage media) separately.

### 4.3. Business Applications

Business applications are designed to accomplish business logic. Specifically, it provides web UI, system configuration, machine learning algorithm management, and data management [19].

1. Three services are proposed, namely, the java application for AIOps, whose content is the implementation of business logic, the ETL application using Spark Stream technology, implemented by Scala, and the AI application providing machine learning capabilities.
2. The spring cloud components are introduced in the java application, such as spring boot, spring gateway, and eureka. Front-end services are written by VUE and deployed on the Nginx server. The front end and java application are decoupled through rest services of the java application.
3. These functions are provided in the java application, which consists of three parts. The first part includes service management, configuration, and web services for integration, including real-time data access and batch data access, alarm data processing, and events. The second part is the management of artificial intelligence models, data management, algorithm configuration, and training of models. The third part is the storage design. The database tables are carefully designed so that data from third parties can be easily integrated into the system.

### 4.4. Machine Learning Module

Machine Learning Algorithms.

The AI service provides implementations of various machine learning algorithms or deep learning algorithms for anomaly detection, classification, and prediction. It also gives the AIOps system the ability to perform the following functions: semi-automatic annotation, feature selection, data normalization, automatic generation of hyperparameters and their optimization, rolling optimization of model hyperparameters, model evaluation, model deployment, and algorithm management.

### 4.5. Design Ideas

The following automatic functions need to be provided. In addition to this, a human intervention interface needs to be provided so that one can review or correct the computational results of the model. Otherwise, the diligence requirements will not be met [20].

1. Automaticity of the model can be reflected in automatic optimization of hyperparameters, automatic updating of the model, and automatic detection and correction of abnormal data.
2. Data competition needs to be considered to avoid AI applications and other applications accessing the same storage medium at the same time, thus mitigating the risk of SPOF.
3. In terms of the response time, the system should give a real-time response to the user. For example, from a technical point of view, the predicted values for the next few hours should be given. In this way, the difference between the actual data and the predicted data can be compared immediately when the actual data are available, rather than performing time-consuming prediction calculations in real-time.
4. Many machine learning algorithms, especially time series algorithms, require high-quality data. For time-series data, the anomalous data must usually be properly processed and the time span between the anomalous data must be strictly determined before computation. In some cases, some auxiliary processes are even required. For example, often two differentiations are done in ARIMA to obtain a stable data set.
5. These algorithms must be configurable so that they can be easily maintained, such as CRUD operations and hyperparameter settings.
6. The deployment management of the model should be included; not only the upgrade of the model, but also the fallback when the model fails.
7. Selection rules for training data, a mechanism for generating data labels, and archiving historical data should all be established. For example, the training data should preferably be balanced rather than biased, and the batch size should also be set to some extent according to the training speed and accuracy requirements of the model.
8. There is no point in telling an algorithm whether it is good or bad without considering the business scenario and the inherent characteristics of the data. In other cases, the most popular algorithm may not be the right algorithm to solve your problem. Instead, you should let the results of the algorithm tell which one is the best. In fact, in many cases, even a simple algorithm may beat a popular algorithm in a given training data, depending on how well the algorithm matches the inherent characteristics of the data.

In order to implement dynamic threshold features for temperature data and to filter the best algorithms, three algorithms, ARIMA, LSTM, and Holt-Winters, are introduced and their performance on the same dataset is compared.

The implementation of ARIMA will be used as an example to show how machine learning algorithms work in a real AIOps project.

### 4.6. Core Implementation of ARIMA

ARIMA is a traditional time-series algorithm that is widely used for forecasting [21]. Considering the fact that the whole process of ARIMA is an important reference for other machine learning algorithms applied to other projects, some main details of ARIMA implementation will be given.

First, from the perspective of the data center room, the temperature of the room is relatively stable within a certain narrow range compared to the wide variation of temperature in different seasons. On the other hand, many data center specifications have their own standards for the temperature of data center rooms, some of which are mandatory.

Three months of temperature data were taken and analyzed to plot the time series. At that time, there was really no sign of seasonality or trend seen. It is clear from the line graph that there was a rapid and sharp drop in data at the beginning of August, and significant fluctuations in data throughout July, which is reasonable because the data center was in the trial installation phase before August.

Finally, after the trial run phase, the data in August were more stable.

**Figure 4:**
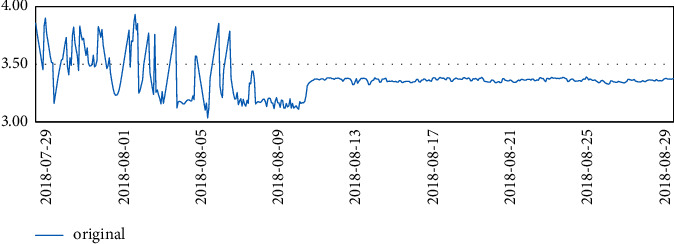
Temperature data.

**Figure 5:**
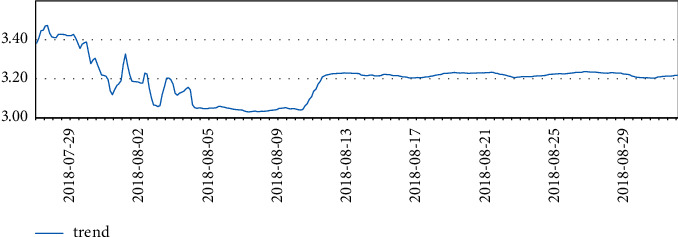
Temperature data.

**Figure 6:**
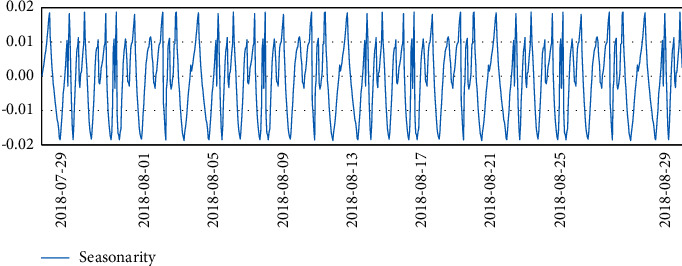
Temperature data.

**Figure 7:**
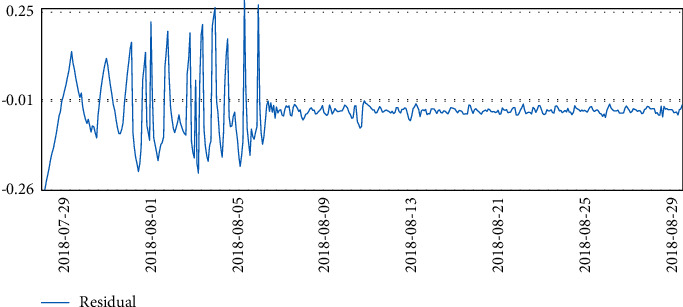
Temperature data.

The temperature data changes are shown in Figures 4–7.

However, it was found that although the stable part of these data, such as the data for August, was used, the ADF test was still unsatisfactory. In particular, the *p* value of ADF was 0.05 and the ADF value of the data was 0.063154, which is greater than 0.05 and is generally not accepted.

The results are as follows:

  test statistic −2.766938
  the *p* value is 0.063154
  # of lags used 21.000000
  number of observations used 816.000000
  critical value (1%) −3.438389
  critical value (5%) −2.865088
  critical value (10%) −2.568660

Therefore, single-difference and double-difference experiments were done separately to verify which result would be better. As a result, the results of double-difference experiments were better than those of single-difference experiments. In addition, triple differencing was also tried. Unfortunately, it was found that there was only a very small boost in ADF, and there was a significant processing delay. Therefore, the recommendation is *d* = 2.

On the other hand, there are other hyperparameters, such as *p* and *q*. One way to obtain the values of these two parameters is to manually use ACF and PACF. Obviously, such a method is not efficient compared to using the auto-ARIMA function to automatically generate the values of *p*, *d*, and *q*. However, in the project, it was found that if the value of *d* was set manually by observing the real behavior of the data line. For example, *d* = 2 was initially set after having a clear understanding of the data. Given the value of *d*, auto-ARIMA can be used to automatically generate *p* and *q* and finally get the best combination of *p*, *d*, and *q*.

Moreover, time series algorithms, such as ARIMA, Holt-Winters, and LSTM, need to classify the training data within a fixed time interval. However, in reality, this is almost an impossible task for various reasons.

The answer is to take the last data as the starting data. Then, re-sort the data in the given time interval. For example,

  Initial data.
  2018/8/19 0:49 23
  2018/8/19 1:53 23.2
  2018/8/19 2:57 23.6
  2018/8/20 3:01 23.6
  2018/8/19 4:01 24
  2018/8/19 5:05 24.4
  2018/8/19 6:09 23.1
  2018/8/19 7:13 23
  2018/8/19 8:17 23.4
  2018/8/19 9:21 22.9
  2018/8/19 10:25 22.9
  2018/8/19 11:29 23.1
  2018/8/19 12:33 23.6

In the abovementioned data, the last data, “2018/8/19 12:33 23.6,” is used as the starting data, then, a fixed time interval is deducted from 2018/8/19 12:33 to calculate the time of the previous data. In this case, the step is 1 hour and the previous time is “2018/8/19 11:33.” Then, the most recent data, “2018/8/19 11:29 23.1,” will be used as the value at “2018/8/19 11:33,” and so on.

Finally, the best combination of hyperparameters was obtained and the prediction was completed, but the processed data are needed to be reversed in order to recover the actual meaning of the data. The reason for this is the double difference operation performed on the initial data.

In terms of the technical specifications of the algorithms, RMSE and MAPE were mainly used to evaluate the performance of LSTM, ARIMA, and Holt-Winters. Interestingly, the top ranking is Holt-Winters rather than LSTM or ARIMA.

### 4.7. Results

ARIMA is not the best algorithm for predicting data center room temperature compared to Holt-Winters, but it performs better than LSTM.

**Figure 8:**
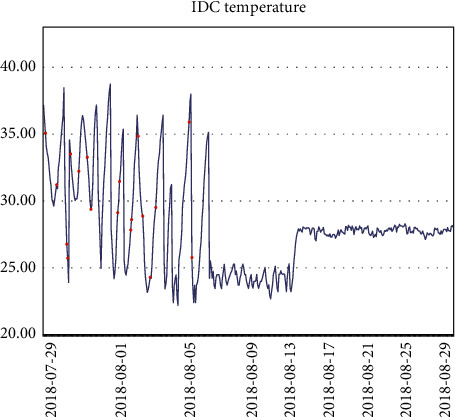
Dynamic threshold graph. ARIMA dynamics.

Those red dots represent the anomalous data; the light blue background is the dynamic threshold based on the temperature values; the dark blue line is the real data; whenever the real data come in, this data are compared with its predicted value. Once the difference value exceeds the current threshold, an alarm is raised and the data are marked as a red exception in the graph and in the system, respectively, as shown in Figure 8.

**Figure 9:**
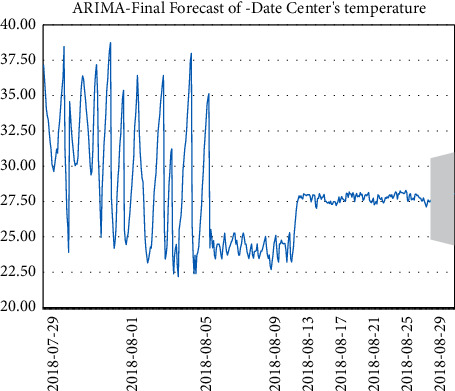
ARIMA prediction diagram.

As shown in Figure 9, the ARIMA prediction chart shows another feature, the prediction function, which can help to do some preparation before an event occurs. In the abovementioned figure, the green line on the far right with the gray background is the prediction line.

Deployment architecture: typically, there are 3 types of deployment options, namely small, standard, and large.

Small deployment means that all the modules are deployed on a single server, which requires corresponding high-performance servers.

**Figure 10:**
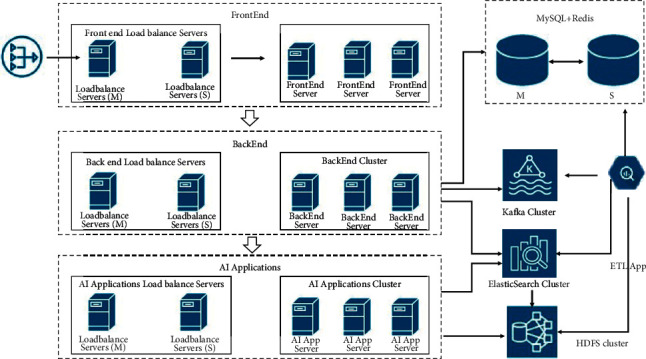
Standard deployment diagram.

Here, the standard deployment diagram is given. Since the standard deployment is scalable, to get the large deployment, only the standard scheme needs to be extended, as shown in Figure 10.

## 5. Conclusion

Many real-world practices have shown that a working architecture or algorithm cannot be directly replicated from another domain due to completely different business scenarios and different data characteristics. In this paper, an AIOps architecture for the data center infrastructure monitoring domain is presented. The details of a proven working architecture and design in practice are presented in terms of core modules such as technical architecture, machine learning algorithms, big data, and business applications. Focusing on the technical part rather than every part of the architectural work, this paper presents a complete and detailed AIOps architecture. AIOps and APM (application performance management IT infrastructure management) systems are rapidly evolving; however, AIOps technology has not made any significant progress in data center infrastructure management. It is designed for data center on-site infrastructure monitoring. The mechanism and architecture design are described by the implementation and real-world performance of a number of different machine learning algorithms. Using data center room temperature data as an example, the test data clearly demonstrate the effectiveness of this architecture. In addition, this architecture can be a useful reference for those who want to know how to implement machine learning algorithms in data center site monitoring systems.

1. This is a productive working architecture. As a time-series database, Elasticsearch is effective and can be used as a time-series database of choice.
2. The most suitable algorithm for the problem is probably the common one, not the most popular one.

In fact, these algorithms should be allowed to compete with each other by running them with the same dataset and the results will tell the winner. Therefore, the algorithm that is best suited to the data and scenario is the best algorithm.

In addition, the research in this paper has some limitations, mainly the following.

### 5.1. AI Governance Capability

As AI models continue to grow, there is a need to continuously monitor these models. Some metrics such as fairness, interpretability, robustness, and transparency should also be taken into account. On the other hand, AI systems themselves need to be governed. Specifically, engineering, development, deployment, and maintenance should be tracked and audited throughout the product lifecycle.

### 5.2. Multidimensional Relevance

In some cases, the accuracy and effectiveness of anomaly detection, especially those predicted by a single feature, are not always that good. Therefore, better results can be obtained if multiple correlated features could be used with other appropriate algorithms, e.g., correlation algorithms. In addition, it is often necessary to identify those relevant features and complete the prediction in one algorithm, e.g., a neuronal network.

However, the problem of how to automatically select features can arise, especially when there are hundreds of features. A reliable approach is to use some automatic function or library to complete the first round of automatic selection. Then, these selected features can be verified by real data or expert experience. Finally, a reasonable feature set can be obtained.

### 5.3. Service Network

Different development languages and frameworks are used, such as Java, Vue, Scala, and Python, but no service grid or other microservice frameworks are used to unify these different services into one layer.

## Figures and Tables

**Table 1:** Power and environmental equipment and indicators.

Components	Items
Power monitoring	Coulometer, distribution switch, UPS, diesel generator, power distribution cabinet, PDU, DC power supply system, battery pack, STS, and ATS

Environment monitoring	Temperature and humidity, precision air conditioning, water leakage, computer room air conditioning, fresh air unit, smog, and harmful gas

Security monitoring	CCTV, access control system, fire extinguisher system, lightening protection detection, and antitheft monitoring

^∗^Network monitoring (Optional)	Router, switch, server, and firewall

**Table 2:** t_devicetypemap.

Name	t_devicetypemap			

Primary key	id			

Foreign key	oriDevType			

Column	Specification	Type	Default value	Remarks

id	Auto-increment 1	bigint unsigned	Nonempty	

oriDevType	Source device type	varchar (64)	Not null	

sDevType	Destination device type	int	Not null	

**Table 3:** t_metetypemap.

Name	t_metetypemap			

Primary key	id			

Foreign key	oriDevType, oriMtType			

Column	Specification	Type	Default value	Remarks

id	Auto-incremented by 1	bigint unsigned	Not null	

oriDevType	Source device type	varchar (64)	Not null	

Metric type (oriMtType)	Source metric type	varchar (64)	Not null	

sDevType	Decomposition device type	int	Not null	

sMtType	Type of decomposition metric	int	Not null	

**Table 4:** Handling of exception data.

Type of anomaly	Policy
Point anomalies ([15], “anomaly detection. a survey”)	Marked as an anomaly
Contextual anomalies ([15], “anomaly detection: a survey”)	Discarded directly and replaced with the most recent data.
Null values	Discarded directly and replaced with the most recent data.
Missing values	Discarded directly and replaced with the closest data.
Duplicate values	When the timestamp, object, and location are identical, they are deleted.

## Data Availability

The dataset can be accessed upon request.
